# Diagnosis and treatment of a patient with severe hepatitis and rash caused by toripalimab: A case report

**DOI:** 10.1097/MD.0000000000044122

**Published:** 2025-08-29

**Authors:** Wei Guo, Yurun Li, Jing Li, Liping Dai, Yuting Jia, Ting Jiang

**Affiliations:** a Department of Pharmacy, Sichuan Shehong People’s Hospital, Shehong, Suining, Sichuan Province, China; b Department of Pharmacy, Clinical Medical College and The First Affiliated Hospital of Chengdu Medical College, Chengdu, Sichuan Province, China.

**Keywords:** exanthema, hepatitis, immune checkpoint inhibitors, multi-viscera, therapy

## Abstract

**Rationale:**

Immune-related adverse events have been frequently reported using immune checkpoint inhibitors. However, these reports are commonly observed in single-organ toxicity. Thus, the compound, multiple, multisite, and multi-organ toxicities are seldom recognized and lack treatment experience, finally leading to severe challenges in the clinical treatment of such patients. More case studies are needed to fully understand the diagnostic and treatment strategies formulti-organ immune-related adverse events. This report aims to share the clinical diagnosis and treatment experience of a patient with nasopharyngeal carcinoma who developed severe hepatitis and rash after treatment with toripalimab.

**Patient concerns:**

A 57-year-old male farmer was diagnosed with nasopharyngeal carcinoma. Ten days after treatment with the first cycle of terprilizumab combined with gemcitabine and cisplatin, the patient was admitted to the hospital for abnormal liver function tests. Two days after admission, the patient developed another rash.

**Diagnoses:**

Based on the patient’s past history, medication history, personal history, and relevant laboratory and imaging examinations, the liver injury and rash for this patient is probable caused by toripalimab.

**Interventions:**

The patient received methylprednisolone sodium succinate injection for anti-inflammatory, tiopronin and magnesium isoglycyrrhizate for hepatoprotective, fluticasone propionate cream and cetirizine for anti-allergic.

**Outcomes:**

The patient’s hepatitis and rash were effectively controlled after treatment, and no reoccurrence was observed during subsequent antitumor therapy.

**Lessons:**

This case proposes that clinician should comprehensively determine the risk of adverse reactions and strengthen medication monitoring to ensure patient safety.

## 1. Introduction

Immune checkpoint inhibitors (ICIs), the monoclonal antibodies, block key mediators imparting immune escape in tumor cells,^[1]^ thereby allowing the immune system to recognize and attack the cancer cells more effectively.^[2]^ In 2011, immunotherapy made key progress in oncology treatment when the first ICIs (ipilimumab) were approved by the FDA for the treatment of melanoma.^[3–5]^ FDA-approved ICIs currently include Programmed Death 1 (PD-1) inhibitors or its ligand Programmed Cell Death-Ligand 1 and cytotoxic T lymphocyte-associated antigen 4.^[6]^ ICIs are one of the effective treatments for many tumors; however, their use may advance to the occurrence of immune-related adverse events (irAEs), affecting tumor treatment and even endangering patients’ lives.^[1]^ All body organs and tissues may be affected by irAEs. The skin, gastrointestinal, endocrine, hepatic, and pulmonary toxicities are more common, while neurological and cardiovascular toxicity are rare.^[7,8]^ Most irAEs occur as a single unit; however, a small number of irAEs may occur in multiple organs simultaneously, thus enhancing clinical treatment challenges.^[9]^ Most of the current recommended guidelines for irAE management are based on a single organ; thus, a specific reference is lacking for the clinical treatment of complex, multiple, multisite, and multi-organ irAEs. This report presents the diagnosis, treatment, and pharmaceutical monitoring of a patient with severe hepatitis and rash after the use of toripalimab, to serve as a guide for the management and treatment of similar cases in the future.

## 2. Case presentation

**Figure 1.**
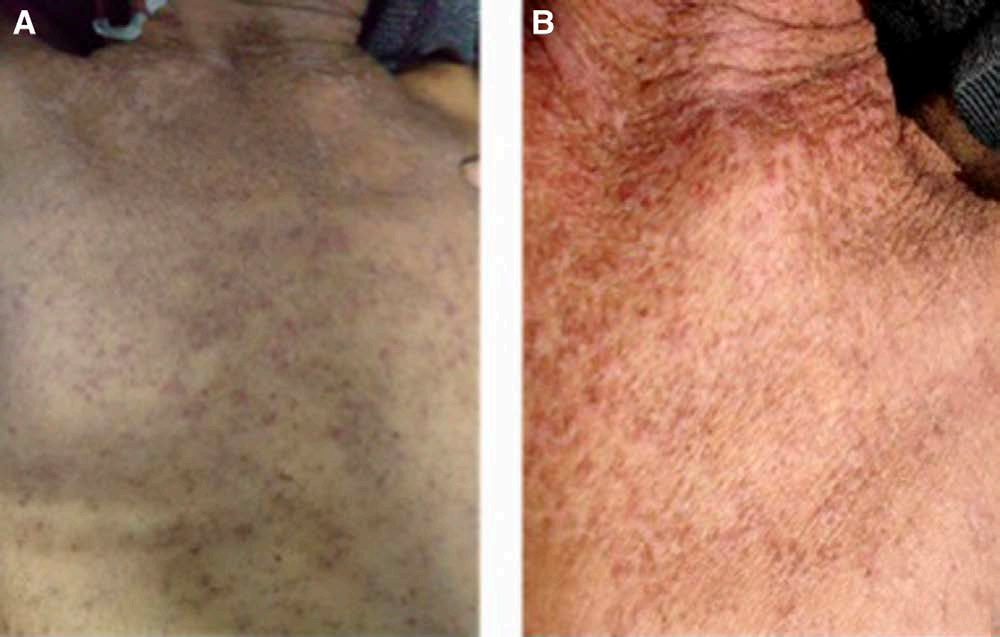
(A and B) Patient’s rash on the 3rd day of admission.

**Figure 2.**
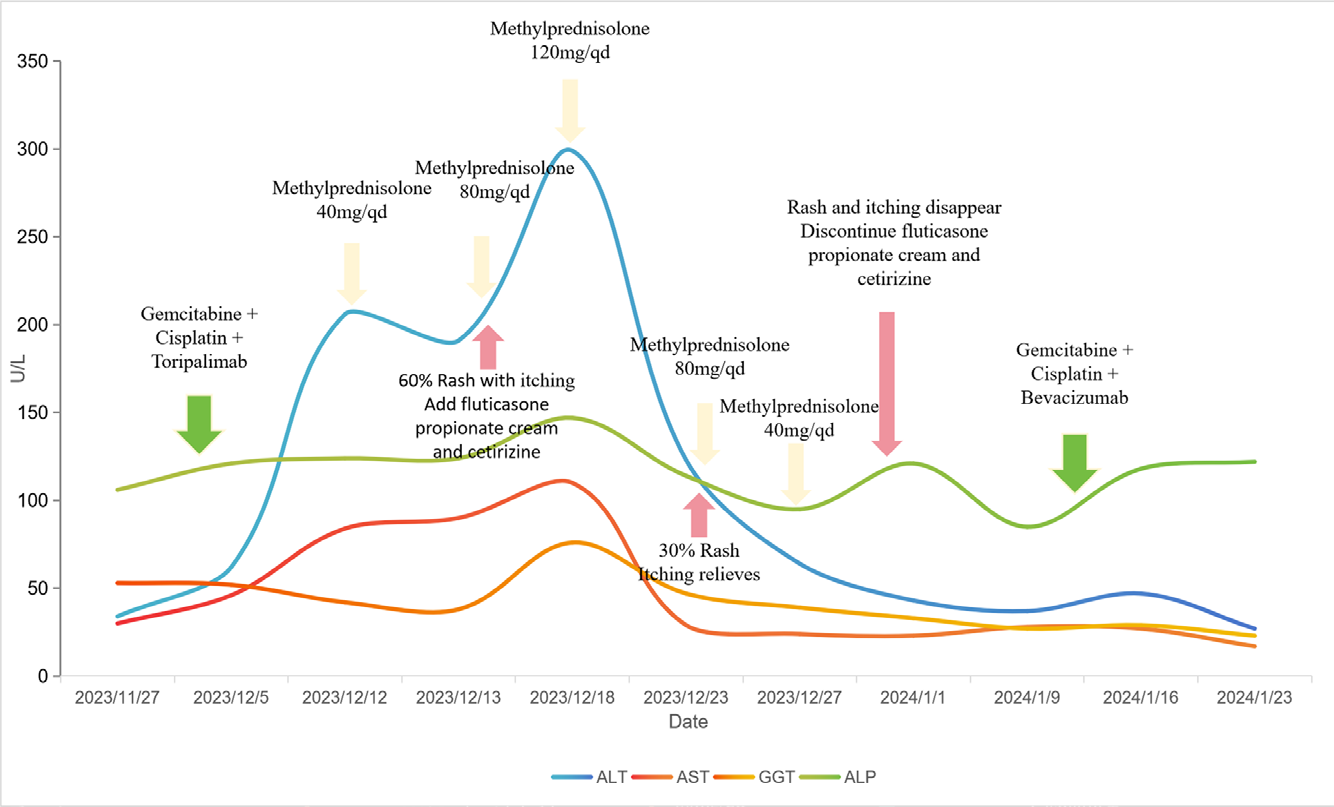
The liver function indices, rash changes, and treatment process of the patients during the treatment period.

A 57-year-old middle-aged male with a body surface area of 1.5 m^2^ and performance score of 1 was admitted with the chief complaint of “left lower limb pain and oral pain” in November 2023. The findings of the image were as follows: the presence of the left gluteus medius and parailiac mass; involvement of the tongue, the right anterior wall of the oral cavity, and bilateral buccal muscles; bilateral cervical perisheath and submandibular multiple lymph nodes showing some slightly larger nodes; the left maxillofacial skin was slightly hyperintense and small, left nasal cavity was swept into a flaky abnormal signal shadow, and left nasal passage was not patent. Based on the pathological results, the provisional diagnosis of sarcomatoid carcinoma was made. Based on the imaging data combined with pathological results, the final diagnosis of oral sarcomatoid carcinoma was made with the tongue, neck lymph nodes, and left iliac metastases stage IV (T4N3M1). The treatment was initiated as follows: on December 2, the first cycle of toripalimab plus gemcitabine plus cisplatin antitumor therapy was performed (toripalimab 240 mg d1 + gemcitabine 1400 mg d1, d8 + cisplatin 30 mg d1–2, 40 mg d3, 21 days a cycle). The patient was readmitted to the hospital on December 12, 2023, owing to “fatigue and loss of appetite,” and no obvious abnormalities were demonstrated through physical examination. Liver function tests indicated the following results: alanine aminotransferase (ALT) 206 U/L, aspartate aminotransferase (AST) 84 U/L, γ-glutamyl transpeptidase 42 U/L, and alkaline phosphatase 124 U/L. The abdominal ultrasonography excluded liver metastases and obstruction. The rest of the renal function and electrolyte tests showed no obvious abnormalities. The patient had no medical history and denied food or drug allergy history. Based on the liver function test results, the patient was immediately treated for hepatoprotective therapy with tiopronin (0.2 g, qd), and administered methylprednisolone sodium succinate injection (40 mg, qd) for anti-inflammatory treatment. Moreover, omeprazole (40 mg, qd) was administered to prevent gastric mucosal lesions owing to hormones. On December 13, 2023, ALT levels were slightly reduced. The patient continued the original treatment regimen and liver function indicators were assessed. The next day, pin-to-miliary size papules and maculopapular rashes were observed densely distributed on approximately 60% of the patient’s limbs and trunk, accompanied by itching without exudation. The observations made for patient’s skin are shown in Figure 1. The physician in charge recommended that the patient undergo a histopathological examination of the skin, but the patient and his family refused. Fluticasone propionate cream was topically used, followed by oral cetirizine (1 mL, qn) for anti-allergic treatment, and an increase in methylprednisolone dose to 80 mg/d. Liver function tests revealed a gradual rise in indicators on December 18, 2023, and 120 mg/d of the methylprednisolone dose was increased again, and magnesium isoglycyrrhizate (200 mg, qd) was added to hepatoprotective therapy. On December 20, 2023, there was a decrease in the patient’s rash, and itching was reduced, and he continued his current treatment. On December 23, 2023, liver function tests demonstrated improvement of the indicators, the patient’s body rash was reduced to approximately 30%, and itching was relieved than before. Methylprednisolone was reduced by 80 mg/d. On December 27, 2023, the liver function indicators improved significantly, and the methylprednisolone dose was reduced to 40 mg/d again. No rashes were observed on December 31, 2023. The skin was smooth, with no obvious pigmentation, and no itching symptoms. Therefore, cetirizine and fluticasone propionate were discontinued. The liver function indicators returned to normal on January 1, 2024, and methylprednisolone tablets (40 mg, qd) were advised, and gradually it was discontinued. The liver function indices, rash changes, and treatment process of the patients during the treatment periodare shown in Figure 2.

## 3. Discussion

### 3.1. Analysis of the causes related to the adverse reactions

#### 3.1.1. Analysis of the liver injury causes

On December 2, 2023, the first cycle of toripalimab in combination with gemcitabine and cisplatin antitumor therapy was administered, and the liver function indices of the patient increased on December 12, 2023. The causes of tumor liver metastasis and liver obstruction were excluded based on the patient’s past history, medication history, personal history, and relevant laboratory and imaging examinations. Cisplatin is primarily excreted by the kidneys. The biliary tract eliminates only a small amount of cisplatin and its degradation products; therefore, the liver injury can be preliminarily ruled out to be caused by cisplatin. Gemcitabine is a cytosine analog with common adverse effects, including the suppression of the bone marrow, fatigue, flu-like symptoms, diarrhea, nausea, gastrointestinal upset, and stomatitis.^[10]^ Transient elevations in serum enzymes are observed during treatment using gemcitabine, which is commonly mild to moderate, asymptomatic, self-limiting, and often resolves spontaneously without discontinuation of treatment.^[10]^ Therefore, it is associated with acute, clinically significant liver injury.^[11]^ Only a few cases of gemcitabine-induced liver injury have been reported, with most occurring in patients with underlying chronic liver disease or extensive liver metastases.^[12,13]^ No previous history of chronic liver disease was noted. The evaluation using imaging excluded liver metastases and obstruction. ALT and AST levels were progressively elevated without the next therapy cycle, thus preliminarily ruling out liver injury owing to gemcitabine. The first domestic PD-1 monoclonal antibody approved in China in 2018 is Toripalimab, which is a PD-1 inhibitor.^[14]^ The reported adverse effects can be observed in immune-related hepatotoxicity. Currently, the immunotherapy-mediated hepatotoxicity (IMH) mechanism is incomprehensible.^[15]^ This may be related to the attack of activated T cells on normal liver tissue, autoantibodies production, and release of inflammatory cytokines by immune cells to mediate tissue immune damage.^[16]^ Hepatotoxicity associated with ICIs demonstrates an increase in the value of liver index parameters, commonly AST and ALT values, with or without an increase in bilirubin.^[17]^ The probability of IMH occurring with PD-1 is 1% to 4%.^[18,19]^ The timing of IMH occurrence is also uncertain and may occur during immunotherapy or several months after the end of treatment.^[20–23]^ The patient developed elevated ALT and AST levels 10 days after the first dose of toripalimab, and the time and symptoms of adverse reactions were consistent with IMH characteristics. The liver injury was categorized as “probable” as an adverse effect of toripalimab using Naranjo’s Adverse Drug Reaction Scale^[24]^ (score of 6).

#### 3.1.2. Analysis of the causes of rashes

On December 14, 2023 (the third day after the abnormal liver function indicators were detected), pinhead to miliary-sized papules and maculopapular rashes were observed densely distributed in approximately 60% of the skin of the patient’s face, neck, and trunk, accompanied by itching, but without exudate. No history of food or drug allergy was found, and the first cycle of antitumor therapy underwent smoothly, without any skin-related adverse reactions. The drugs used were tiopronin, methylprednisolone sodium succinate, and omeprazole. Adverse skin reactions caused by the 3 drugs are reported less in the literature. A review of skin-related adverse reactions of proton pump inhibitors suggested that most were IgE-mediated immediate allergic reactions.^[25]^ Most of the adverse reactions owing to methylprednisolone were immediate hypersensitivity reactions, mostly occurring on the day of administration.^[26]^ Therefore, the rash was preliminarily ruled out to be related to the drug use during this hospitalization and was suspected to be an adverse reaction owing to the previous cycle of antitumor therapy. Mostly type I anaphylaxis occurs by conjugated cisplatin, mediated by IgE, and commonly occurs after several courses of chemotherapy, primarily manifesting as itching, urticaria, facial swelling, bronchospasm, and hypotension.^[27]^ Additionally, skin-related adverse reactions owing to cisplatin have been occasionally reported. Therefore, the rash is preliminarily ruled out to be caused by cisplatin. Therash is a common skin-related adverse effect of gemcitabine. It occurs in approximately 25% of patients, accompanied by pruritus in 10% of patients. Rash is a non-dose-limiting toxicity^[28]^; however, symptoms are generally mild.^[29,30]^ Liu Xia et al demonstrated that the median time to rash with gemcitabine was 6 (1–18) days, and duration ranged from 5 to 20 days in 36 cases of drug-induced rash caused by gemcitabine, all of which occurred when gemcitabine was first used. The rash primarily occurs on the chest, lower back, armpits, groin, perineum, and buttocks.^[31]^ The time of onset and distribution characteristics of the rash were consistent with those reported in the literature; therefore, the possibility of gemcitabine causing the rash could not be excluded. Simultaneously, the most common adverse events caused by ICIs are cutaneous and commonly occur first.^[32]^ These events commonly occur weeks to months after the initiation of immunotherapy; however, they can occur at any time. Among them, maculopapular rash commonly occurs within the first 3 to 6 weeks after ICI therapy initiation.^[1]^ Predominately, the extensor sides of the trunk and extremities are affected, while the head, palms, and soles are generally spared.^[33]^ Based on the package insert for toripalimab, approximately 10.2% of patients receiving toripalimab plus chemotherapy demonstrated immune-related cutaneous adverse reactions, including 4.6, 3.1, and 2.5% for grades 1, 2, and 3, respectively, with a median time to occurrence of 1.2 r (0.1–23.1) months and a median duration of 2.2 (0.1–35.2) months. Therefore, the occurrence of rash is also associated with toripalimab. The association between toripalimab and gemcitabine causing a “rash” in patients was “probable” using Naranjo’s Adverse Drug Reaction Scale^[24]^ (score of 6). It is speculated that the rash developed was probably immune-related after IMH, At the same time, the rash occurs when the ALT is better than before, considering that liver injury in this patient is related to immunotherapy drugs. Simultaneously, the incidence and severity of rash using toripalimab combined with gemcitabine and cisplatin have been higher than those using gemcitabine and cisplatin in the treatment of recurrent or metastatic nasopharyngeal carcinoma.^[34]^ Therefore, the rash in this patient is “probable”caused by toripalimab; however, the superimposed effect of gemcitabine cannot be excluded. Because the patient did not undergo a skin histopathological examination, there are several limitations in the conclusion of this study. For atypical, severe, and recurrent rashes, skin biopsy can help detect drug-related factors early.^[35]^

### 3.2. Adverse effects treatment decision-making

The patient had grade G2 hepatotoxicity at admission according to the United States National Cancer Institute’s Adverse Event Evaluation Criteria version 5.0 (NCI-CTCAEv5.0). According to the National Comprehensive Cancer Network (NCCN)^[36]^ and Chinese Society of Clinical Oncology (CSCO)^[37]^ guidelines, it is recommended to suspend immune checkpoint inhibitor therapy. Simultaneously, 0.5–1 mg/(kg d) of prednisone equivalent dose hormone therapy is recommended for G2 hepatotoxicity. The initial treatment regimen was methylprednisolone 40 mg daily for this patient. Approximately 60% of the patient’s limbs and trunk were densely covered with pinhead-to-miliary size papules and maculopapular rashes with itching and no exudate after 2 days of treatment, which could be assessed as G3 cutaneous toxicity according to NCI-CTCAEv5.0. Strong glucocorticoids are recommended for external use, according to the NCCN and CSCO guidelines, and simultaneously, an equivalent dose of 0.5–1 mg/(kg d) prednisone should be administered for hormone therapy. If there is no improvement, the dose can be increased to 2 mg/(kg d), and antihistamines can be administered if rashes are accompanied by itching. Subsequently, this patient was treated with an increase in methylprednisolone dose to 120 mg/d, combined with topical application of fluticasone propionate cream and oral cetirizine. Clinical pharmacists considered that long-term use of high-dose corticosteroids might cause adverse reactions in multiple organ systems, even inducing infection^[38–40]^; therefore, glucocorticoids are recommended to be tapered to discontinuation assuming that the immune-related toxicity does not worsen, and clinicians adopt it.

The IrAEs management comprises using immunosuppressants, currently known as glucocorticoids, cytokine antibodies, tumor necrosis factor-α inhibitors, gamma globulin, and rituximab. Both NCCN and CSCO current guidelines recommend 2 mg/(kg d) methylprednisolone as the maximum dose of glucocorticoids for the treatment of single-organ irAEs. However, the management of multi-organ IrAEs has been rarely reported. Deng C et al^[41]^ and Hu X et al^[42]^ concluded that the early application of low-dose glucocorticoids and dose adjustment through the evolution of multi-organ IrAEs is is the key treatment for the immune-related multi-organ injury. If high-dose corticosteroids are ineffective, other immunosuppressants may be considered. This is consistent with our approach. The type and severity of irAEs, the initial dose of glucocorticoids, and the patient’s response to treatment affected the total duration of glucocorticoid reduction. The tapering of glucocorticoids should be slow, generally over 4 to 6 weeks. The patient should be closely observed clinically during tapering, as excessively rapid tapering can lead to irAE exacerbation or the appearance of new symptoms. Basically, oral prednisone is reduced by 10 mg every 3 to 7 days.^[37]^

### 3.3. Whether the patient can restart immunotherapy

According to the NCCN and CSCO guidelines, this patient met G3 hepatotoxicity and G3-4 cutaneous toxicity. The guidelines vary as to whether toripalimab can be reinitiated in this patient, as shown in Table 1. The literature was reviewed. A meta-analysis of the safety and efficacy of rechallenge of ICIs after irAEs in patients with cancer demonstrated that the incidence of adverse and serious adverse reactions after rechallenge of immunosuppressants was 34.2% and 11.7%, respectively. The incidence of adverse effects of immune rechallenge was higher than that of initial treatment; however, that of serious adverse effects was similar.^[43]^ A higher risk of recurrence of adverse effects after immune rechallenge was observed by Kartolo et al in patients who developed grade 3 irAEs on initial treatment.^[44]^ A quarter and a third of patients demonstrated relapse with the same irAEs after the first episode of irAEs in another retrospective study, which was rechallenged with immunosuppressants, and the IMH recurrence rate was higher than that of other irAEs.^[45]^ Based on relevant guidelines and literature reports, the clinical pharmacist believes that the patient is extremely likely to develop the same or other irAEs after re-treatment with toripalimab. Simultaneously, the patient had multisite irAEs adverse reactions with liver damage and severe rash. Moreover, the adverse reactions were all graded G3 with high severity, with a high risk of the reuse of ICIs. Therefore, the patient was recommended to discontinue using immunotherapy, which was adopted by the clinicians.

**Table 1:** Recommendations for restarting immune checkpoint inhibitors (ICIs).

Type/grade of adverse reactions	Guide	Recommended opinion
Hepatotoxicity/G3 grade	NCCN	Immunotherapy is suspended, and in cases of severe or life-threatening (grade 4) hepatitis, permanent discontinuation is required
	CSCO	ICIs are recommended to be discontinued until the prednisone dose is reduced to ≤10 mg/d and hepatotoxicity is ≤grade 1, and re-ICIs can be considered
Cutaneous toxicity (macules/papules)/G3-4 grade	NCCN	Immunotherapy may be suspended until symptoms have resolved to grade 1, and then re-ICIs may be considered
	CSCO	

CSCO = Chinese Society of Clinical Oncology, ICIs = immune checkpoint inhibitors, NCCN = National Comprehensive Cancer Network.

### 3.4. Follow-up visits

After the patient’s discharge, the dose of prednisone was reduced by 12 mg/wk until discontinuation of the drug (19 days from January 1, 2024, to January 19, 2024). Following this, from January 11, 2024, to January 13, 2024, the antitumor therapy was administered using bevacizumab in combination with gemcitabine and cisplatin (bevacizumab 300 mg d1 + gemcitabine 1400 mg d1, d8 + cisplatin 30 mg d1–240 mg d3, 21 days a cycle). No abnormal liver function or rash adverse reactions were observed.

## 4. Limitations

A limitation of this case study is that no skin histopathology was performed after the patient developed a rash, which leads to a lack of reliable pathological evidence to support the occurrence of the rash and the use of tolizumab. In future similar case studies, we will improve the examination of skin histopathology to clarify the type and cause of the rash.

## 5. Conclusions

We reported a rare multi-organ irAEs, For this case, we not only investigated the association between the occurrence of severe hepatitis and rash and ICIs, but also assessed the treatment formulation and alteration of the diagnosis and treatment plan for multi-organ irAEs. In addition, we presented a comprehensive demonstration of whether ICIs treatment could be reinitiated in the future.

This case emphasizes the need to comprehensively assess the risk of adverse reactions and strengthen medication monitoring. Early diagnosis and intervention are crucial for the prognosis of irAEs.

## Author contributions

**Conceptualization:** Wei Guo.

**Data curation:** Liping Dai, Yuting Jia.

**Writing – original draft:** Wei Guo, Yurun Li.

**Writing – review & editing:** Jing Li, Ting Jiang.
